# 1β,15α-Dihy­droxy-16α,17-ep­oxy­pregn-4-ene-3,20-dione

**DOI:** 10.1107/S1600536813005023

**Published:** 2013-02-28

**Authors:** Yan-Bing Shen, Yi-Bo Wang, Jian-Mei Luo, Min Wang

**Affiliations:** a Key Laboratory of Industrial Fermentation Microbiology (Tianjin University of Science and Technology), Ministry of Education, College of Biotechnology, Tianjin University of Science and Technology, Tianjin 300457, People’s Republic of China

## Abstract

The title mol­ecule, C_21_H_28_O_5_, is composed of three six-membered rings (*A*/*B*/*C*) and a five-membered ring (*D*). Ring *A* adopts a 1α-sofa conformation, while rings *B* and *C* adopt chair conformations. Cyclo­pentane ring *D* adopts a 14α-envelope conformation. In the crystal, O—H⋯O hydrogen bonds lead to the formation of ribbons running along the *a* axis. The structure is further consolidated by C—H⋯O inter­actions, which link the molecules head-to-tail into ribbons along the *a* axis.

## Related literature

For background to 16α,17α-ep­oxy­progesterone, see: Breskvar *et al.* (1995 ▶); Zhou *et al.* (2009 ▶). For the crystal structure of a related compound, see: Nie *et al.* (2005 ▶).
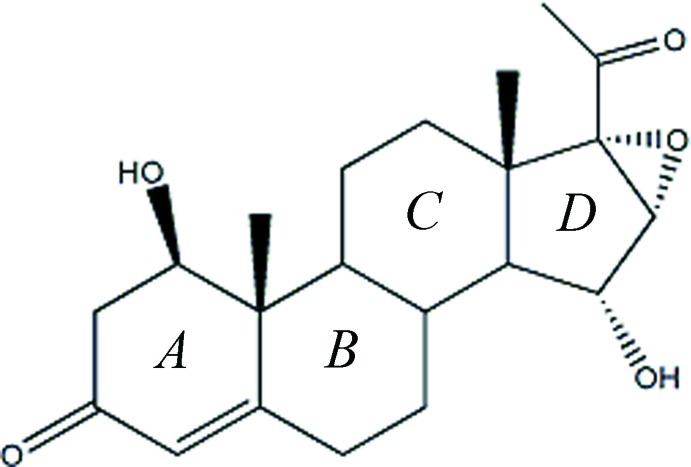


## Experimental

### 

#### Crystal data

C_21_H_28_O_5_
*M* *_r_* = 360.43
Orthorhombic, 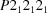

*a* = 7.6372 (10) Å
*b* = 13.7067 (16) Å
*c* = 17.083 (2) Å
*V* = 1788.3 (4) Å^3^
*Z* = 4
Mo *K*α radiation
μ = 0.09 mm^−1^
*T* = 113 K
0.22 × 0.18 × 0.14 mm

#### Data collection

Rigaku Saturn 724CCD diffractometer
Absorption correction: multi-scan (*CrystalClear*; Rigaku, 2005 ▶) *T*_min_ = 0.980, *T*_max_ = 0.987
18889 measured reflections
4237 independent reflections
3529 reflections with *I* > 2σ(*I*)
*R*_int_ = 0.062

#### Refinement

*R*[*F* ^2^ > 2σ(*F* ^2^)] = 0.043
*wR*(*F* ^2^) = 0.077
*S* = 0.94
4237 reflections
246 parameters
H atoms treated by a mixture of independent and constrained refinement
Δρ_max_ = 0.20 e Å^−3^
Δρ_min_ = −0.23 e Å^−3^

### 

Data collection: *CrystalClear* (Rigaku, 2005 ▶); cell refinement: *CrystalClear*; data reduction: *CrystalClear*; program(s) used to solve structure: *SHELXS97* (Sheldrick, 2008 ▶); program(s) used to refine structure: *SHELXL97* (Sheldrick, 2008 ▶); molecular graphics: *SHELXTL* (Sheldrick, 2008 ▶); software used to prepare material for publication: *SHELXTL*.

## Supplementary Material

Crystal structure: contains datablock(s) global, I. DOI: 10.1107/S1600536813005023/pv2620sup1.cif

Structure factors: contains datablock(s) I. DOI: 10.1107/S1600536813005023/pv2620Isup2.hkl

Additional supplementary materials:  crystallographic information; 3D view; checkCIF report

## Figures and Tables

**Table 1:** Hydrogen-bond geometry (Å, °)

*D*—H⋯*A*	*D*—H	H⋯*A*	*D*⋯*A*	*D*—H⋯*A*
O1—H1⋯O4^i^	0.88 (2)	2.06 (2)	2.929 (2)	168
O3—H3⋯O5^ii^	0.86 (2)	1.95 (2)	2.767 (2)	159
C1—H1*A*⋯O3^i^	1.00	2.59	3.527 (2)	157
C6—H6*A*⋯O1^ii^	0.99	2.59	3.527 (2)	158
C12—H12*B*⋯O3^iii^	0.99	2.52	3.468 (2)	161
C21—H21*B*⋯O2^iv^	0.98	2.53	3.501 (2)	170

Symmetry codes: (i) 
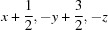
; (ii) 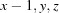; (iii) 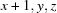; (iv) 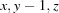.

**Fig. 1.**
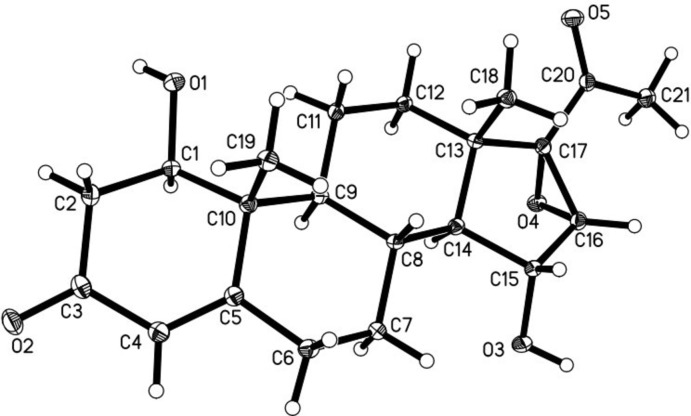
Perspective view of the title compound with 18% probability ellipsoids

**Fig. 2.**
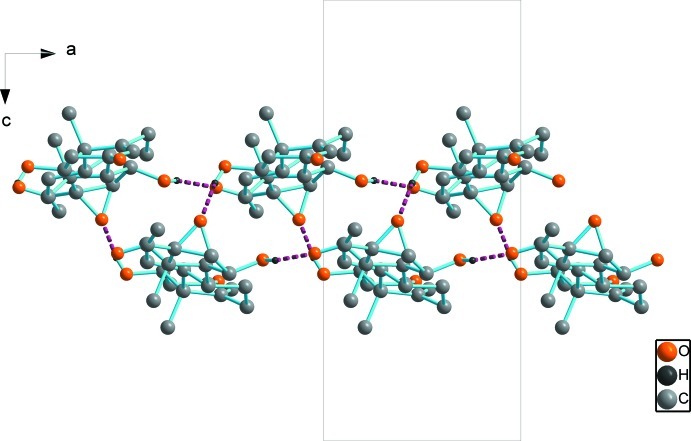
A view of the unit cell packing of the title compound showing intermolecular O—H···O hydrogen bonds forming ribbons of molecules running along the a-axis.

## supplementary crystallographic information

### Comment

16α,17α-Epoxyprogesterone (EP) is an important intermediate for many hormone based pharmaceuticals, such as hydrocortisone and megestrol, produced through 11α-hydroxylation by microorganisms in the industry (Zhou *et al.*, 2009; Breskvar *et al.*, 1995).

The bond distances and angles in the title compound (Fig. 1) agree very well with the corresponding bond distances and angles reported in a closely related compound (Nie *et al.*, 2005). The title compound has three six-membered rings (A/B/C) and one five-membered rings (D). Ring A has a 1α-sofa conformation. Rings B and C adopt chair conformations, while the cyclopentane ring D adopts a 14α-envelope conformation. The 1-hydroxy is in β and 15-hydroxy in α configuration. In the crystal packing (Fig. 2 & Tab 1), there are intermolecular hydrogen bonds O3—H3···O5 and O1—H1···O4 which stabilize the structure and contribute to the formation of one-dimensional ribbons running along the *a*-axis. The structure is further consolidated by intermolecular hydrogen bonding interactions of the tyoe C—H···O (Tab. 1).

### Experimental

*Colletotrichum lini* AS3. 4486 was obtained from Institute of Microbiology, Chinese Academy of Sciences. The strain was cultivated in shake flasks in two stages. Firstly, mycelium was grown on seed medium (glucose 30 g/*L*, corn steep liquor 10 g/*L*, pH 7.0) for 72 h on a rotary shaker (200 r/min) at 298 K. At the second stage, 10% (*v*/*v*) of the first mycelium obtained were added to the growing medium containing (g/l): glucose 30, corn steep liquor 10, soy meal 10, NaNO_3_ 2, KH_2_PO_4_ 1, K_2_HPO_4_ 2, MgSO_4_.7H_2_O 0.5, KCl 0.5, FeSO_4_.7H_2_O 0.02 (pH 7.0) and incubated for 24 h at the same conditions. Thereafter 50 mg of the 16α,17α-epoxyprogesterone dissolved in 1 ml of ethanol was added to the culture after 24 h for growth and the reaction was allowed to proceed for 72 h. The mycelium was then removed by filteration. The biomass and the broth were extracted separately with EtOAc. All extracts were combined and dried (anhydr. Na_2_SO_4_). The solvents after filtration were evaporated under reduced pressure. The crude extracts were purified by Si gel column using dichloromethane/ether/methanol (25:2:1, *v*/*v*/v). The white powder was diffused with n-hexane/acetone at room temperature. Colourless prismatic crystals suitable for X-ray analysis were obtained.

### Refinement

The hydroxyl H atoms were located from difference Fourier maps and refined freely. The H atoms bonded to C atoms were positioned geometrically and refined using a riding model, with C—H = 0.95, 0.98, 0.99, 1.00 Å, for aryl, methyl, methylene and methyne H-atoms, respectively. The *U*_iso_(H) were allowed at 1.5*U*_eq_(C methyl) or 1.2*U*_eq_(C non-methyl). In the absence of sufficient anomalous dispersion effects in diffraction measurements, an absolute structure was not determined 1815 Friedel pairs were not merged.

### Crystal data

**Table:**

C_21_H_28_O_5_	*F*(000) = 776
*M**_r_* = 360.43	*D*_x_ = 1.339 Mg m^−^^3^
Orthorhombic, *P*2_1_2_1_2_1_	Mo *K*α radiation, λ = 0.71073 Å
Hall symbol: P 2ac 2ab	Cell parameters from 4859 reflections
*a* = 7.6372 (10) Å	θ = 1.9–27.9°
*b* = 13.7067 (16) Å	µ = 0.09 mm^−^^1^
*c* = 17.083 (2) Å	*T* = 113 K
*V* = 1788.3 (4) Å^3^	Prism, colorless
*Z* = 4	0.22 × 0.18 × 0.14 mm

### Data collection

**Table:**

Rigaku Saturn 724CCD diffractometer	4237 independent reflections
Radiation source: rotating anode	3529 reflections with *I* > 2σ(*I*)
Multilayer monochromator	*R*_int_ = 0.062
Detector resolution: 14.22 pixels mm^-1^	θ_max_ = 27.9°, θ_min_ = 1.9°
ω scans	*h* = −9→9
Absorption correction: multi-scan (*CrystalClear*; Rigaku, 2005)	*k* = −18→18
*T*_min_ = 0.980, *T*_max_ = 0.987	*l* = −22→22
18889 measured reflections	

### Refinement

**Table:**

Refinement on *F*^2^	Primary atom site location: structure-invariant direct methods
Least-squares matrix: full	Secondary atom site location: difference Fourier map
*R*[*F*^2^ > 2σ(*F*^2^)] = 0.043	Hydrogen site location: inferred from neighbouring sites
*wR*(*F*^2^) = 0.077	H atoms treated by a mixture of independent and constrained refinement
*S* = 0.94	*w* = 1/[σ^2^(*F*_o_^2^) + (0.032*P*)^2^] where *P* = (*F*_o_^2^ + 2*F*_c_^2^)/3
4237 reflections	(Δ/σ)_max_ < 0.001
246 parameters	Δρ_max_ = 0.20 e Å^−^^3^
0 restraints	Δρ_min_ = −0.23 e Å^−^^3^

### Special details

**Table:**

Geometry. All e.s.d.'s (except the e.s.d. in the dihedral angle between two l.s. planes) are estimated using the full covariance matrix. The cell e.s.d.'s are taken into account individually in the estimation of e.s.d.'s in distances, angles and torsion angles; correlations between e.s.d.'s in cell parameters are only used when they are defined by crystal symmetry. An approximate (isotropic) treatment of cell e.s.d.'s is used for estimating e.s.d.'s involving l.s. planes.
Refinement. Refinement of *F*^2^ against ALL reflections. The weighted *R*-factor *wR* and goodness of fit *S* are based on *F*^2^, conventional *R*-factors *R* are based on *F*, with *F* set to zero for negative *F*^2^. The threshold expression of *F*^2^ > σ(*F*^2^) is used only for calculating *R*-factors(gt) *etc*. and is not relevant to the choice of reflections for refinement. *R*-factors based on *F*^2^ are statistically about twice as large as those based on *F*, and *R*- factors based on ALL data will be even larger.

### Fractional atomic coordinates and isotropic or equivalent isotropic displacement parameters (Å^2^)

**Table:**

	*x*	*y*	*z*	*U*_iso_*/*U*_eq_	
O1	0.49950 (15)	0.92527 (9)	0.12185 (7)	0.0224 (3)	
H1	0.551 (3)	0.9592 (16)	0.0847 (13)	0.057 (8)*	
O2	0.03424 (16)	1.15599 (9)	0.13838 (7)	0.0277 (3)	
O3	−0.19159 (16)	0.52524 (9)	0.08958 (7)	0.0229 (3)	
H3	−0.255 (3)	0.4733 (15)	0.0906 (13)	0.045 (7)*	
O4	0.12518 (14)	0.44448 (8)	0.00189 (6)	0.0185 (3)	
O5	0.54475 (15)	0.38806 (9)	0.07485 (7)	0.0263 (3)	
C1	0.3139 (2)	0.94276 (12)	0.11529 (10)	0.0166 (4)	
H1A	0.2810	0.9395	0.0587	0.020*	
C2	0.2779 (2)	1.04568 (12)	0.14471 (11)	0.0206 (4)	
H2A	0.3232	1.0520	0.1988	0.025*	
H2B	0.3420	1.0928	0.1114	0.025*	
C3	0.0870 (2)	1.07134 (13)	0.14422 (9)	0.0199 (4)	
C4	−0.0321 (2)	0.98930 (13)	0.15609 (9)	0.0193 (4)	
H4	−0.1544	1.0019	0.1549	0.023*	
C5	0.0208 (2)	0.89693 (12)	0.16862 (9)	0.0160 (4)	
C6	−0.1108 (2)	0.82234 (12)	0.19573 (10)	0.0190 (4)	
H6A	−0.2299	0.8501	0.1903	0.023*	
H6B	−0.0909	0.8088	0.2520	0.023*	
C7	−0.1019 (2)	0.72628 (12)	0.15031 (10)	0.0172 (4)	
H7A	−0.1793	0.6776	0.1755	0.021*	
H7B	−0.1440	0.7368	0.0962	0.021*	
C8	0.0854 (2)	0.68738 (12)	0.14825 (9)	0.0143 (3)	
H8	0.1243	0.6739	0.2031	0.017*	
C9	0.2095 (2)	0.76441 (12)	0.11175 (9)	0.0140 (4)	
H9	0.1575	0.7811	0.0597	0.017*	
C10	0.2117 (2)	0.86252 (12)	0.15956 (9)	0.0152 (4)	
C11	0.3945 (2)	0.72402 (12)	0.09401 (10)	0.0171 (4)	
H11A	0.4567	0.7714	0.0603	0.020*	
H11B	0.4601	0.7192	0.1438	0.020*	
C12	0.3979 (2)	0.62358 (12)	0.05362 (9)	0.0161 (4)	
H12A	0.3527	0.6297	−0.0005	0.019*	
H12B	0.5199	0.5994	0.0508	0.019*	
C13	0.2860 (2)	0.55152 (12)	0.09924 (9)	0.0141 (4)	
C14	0.0983 (2)	0.59318 (11)	0.10095 (9)	0.0145 (4)	
H14	0.0705	0.6116	0.0457	0.017*	
C15	−0.0208 (2)	0.50721 (12)	0.12027 (10)	0.0164 (4)	
H15	−0.0263	0.4967	0.1781	0.020*	
C16	0.0699 (2)	0.42251 (12)	0.08075 (9)	0.0171 (4)	
H16	0.0394	0.3540	0.0956	0.020*	
C17	0.2552 (2)	0.44928 (12)	0.06428 (9)	0.0154 (4)	
C18	0.3623 (2)	0.53329 (13)	0.18182 (9)	0.0185 (4)	
H18A	0.2983	0.4798	0.2070	0.028*	
H18B	0.3505	0.5926	0.2134	0.028*	
H18C	0.4863	0.5158	0.1774	0.028*	
C19	0.2903 (2)	0.84768 (12)	0.24259 (9)	0.0185 (4)	
H19A	0.2864	0.9095	0.2714	0.028*	
H19B	0.4121	0.8258	0.2380	0.028*	
H19C	0.2221	0.7984	0.2708	0.028*	
C20	0.3939 (2)	0.37170 (12)	0.05503 (9)	0.0171 (4)	
C21	0.3414 (2)	0.27630 (13)	0.02084 (11)	0.0235 (4)	
H21A	0.3040	0.2858	−0.0335	0.035*	
H21B	0.2445	0.2488	0.0513	0.035*	
H21C	0.4412	0.2314	0.0222	0.035*	

### Atomic displacement parameters (Å^2^)

**Table:**

	*U*^11^	*U*^22^	*U*^33^	*U*^12^	*U*^13^	*U*^23^
O1	0.0168 (7)	0.0216 (7)	0.0286 (7)	−0.0011 (6)	0.0030 (6)	0.0020 (6)
O2	0.0319 (8)	0.0174 (7)	0.0339 (7)	0.0057 (6)	0.0032 (6)	0.0027 (6)
O3	0.0141 (7)	0.0206 (7)	0.0341 (7)	−0.0022 (6)	−0.0049 (6)	−0.0006 (6)
O4	0.0173 (6)	0.0210 (6)	0.0172 (6)	−0.0004 (5)	−0.0026 (5)	−0.0010 (5)
O5	0.0169 (7)	0.0224 (7)	0.0397 (8)	0.0009 (6)	−0.0023 (6)	−0.0068 (6)
C1	0.0127 (9)	0.0166 (9)	0.0206 (9)	0.0005 (8)	0.0010 (7)	0.0009 (8)
C2	0.0225 (10)	0.0152 (9)	0.0240 (9)	−0.0014 (8)	−0.0014 (8)	−0.0005 (8)
C3	0.0267 (10)	0.0183 (9)	0.0147 (8)	0.0034 (8)	0.0010 (8)	−0.0016 (8)
C4	0.0188 (9)	0.0207 (9)	0.0186 (9)	0.0012 (8)	−0.0014 (7)	−0.0028 (8)
C5	0.0181 (9)	0.0165 (9)	0.0135 (8)	−0.0005 (8)	0.0007 (7)	−0.0042 (7)
C6	0.0155 (9)	0.0195 (9)	0.0218 (9)	0.0000 (8)	0.0016 (7)	−0.0014 (8)
C7	0.0153 (9)	0.0156 (8)	0.0209 (8)	0.0010 (7)	−0.0006 (8)	0.0016 (7)
C8	0.0116 (8)	0.0155 (8)	0.0159 (8)	0.0012 (7)	−0.0017 (7)	0.0010 (7)
C9	0.0127 (9)	0.0153 (9)	0.0142 (8)	0.0006 (7)	−0.0001 (7)	0.0006 (7)
C10	0.0144 (9)	0.0153 (9)	0.0160 (8)	0.0003 (7)	0.0001 (7)	−0.0008 (7)
C11	0.0164 (9)	0.0145 (8)	0.0204 (8)	−0.0021 (7)	0.0029 (8)	0.0013 (7)
C12	0.0130 (9)	0.0153 (8)	0.0198 (8)	−0.0005 (8)	0.0017 (7)	−0.0010 (7)
C13	0.0130 (9)	0.0145 (8)	0.0148 (8)	0.0007 (7)	−0.0008 (7)	0.0000 (7)
C14	0.0140 (9)	0.0154 (8)	0.0140 (8)	−0.0008 (7)	−0.0011 (7)	0.0003 (7)
C15	0.0128 (9)	0.0164 (9)	0.0199 (8)	−0.0006 (7)	−0.0002 (7)	0.0011 (7)
C16	0.0184 (10)	0.0160 (9)	0.0167 (8)	−0.0019 (7)	−0.0010 (7)	0.0032 (7)
C17	0.0158 (9)	0.0156 (9)	0.0146 (8)	0.0006 (8)	−0.0018 (7)	0.0014 (7)
C18	0.0181 (10)	0.0188 (9)	0.0187 (8)	0.0008 (8)	−0.0012 (7)	0.0010 (7)
C19	0.0186 (10)	0.0194 (9)	0.0176 (9)	0.0012 (8)	−0.0004 (7)	−0.0017 (7)
C20	0.0190 (10)	0.0150 (9)	0.0173 (8)	0.0006 (8)	0.0025 (8)	0.0018 (7)
C21	0.0213 (11)	0.0182 (10)	0.0310 (10)	−0.0003 (8)	0.0010 (8)	−0.0023 (8)

### Geometric parameters (Å, º)

**Table:**

O1—C1	1.4421 (19)	C9—C10	1.573 (2)
O1—H1	0.88 (2)	C9—H9	1.0000
O2—C3	1.232 (2)	C10—C19	1.554 (2)
O3—C15	1.4270 (19)	C11—C12	1.540 (2)
O3—H3	0.86 (2)	C11—H11A	0.9900
O4—C16	1.4434 (19)	C11—H11B	0.9900
O4—C17	1.4583 (19)	C12—C13	1.521 (2)
O5—C20	1.2213 (19)	C12—H12A	0.9900
C1—C2	1.523 (2)	C12—H12B	0.9900
C1—C10	1.546 (2)	C13—C17	1.541 (2)
C1—H1A	1.0000	C13—C14	1.543 (2)
C2—C3	1.499 (2)	C13—C18	1.547 (2)
C2—H2A	0.9900	C14—C15	1.525 (2)
C2—H2B	0.9900	C14—H14	1.0000
C3—C4	1.461 (2)	C15—C16	1.511 (2)
C4—C5	1.346 (2)	C15—H15	1.0000
C4—H4	0.9500	C16—C17	1.488 (2)
C5—C6	1.507 (2)	C16—H16	1.0000
C5—C10	1.540 (2)	C17—C20	1.509 (2)
C6—C7	1.530 (2)	C18—H18A	0.9800
C6—H6A	0.9900	C18—H18B	0.9800
C6—H6B	0.9900	C18—H18C	0.9800
C7—C8	1.527 (2)	C19—H19A	0.9800
C7—H7A	0.9900	C19—H19B	0.9800
C7—H7B	0.9900	C19—H19C	0.9800
C8—C14	1.526 (2)	C20—C21	1.487 (2)
C8—C9	1.550 (2)	C21—H21A	0.9800
C8—H8	1.0000	C21—H21B	0.9800
C9—C11	1.548 (2)	C21—H21C	0.9800
			
C1—O1—H1	107.2 (14)	H11A—C11—H11B	107.5
C15—O3—H3	111.2 (14)	C13—C12—C11	109.95 (13)
C16—O4—C17	61.72 (10)	C13—C12—H12A	109.7
O1—C1—C2	107.82 (14)	C11—C12—H12A	109.7
O1—C1—C10	109.88 (13)	C13—C12—H12B	109.7
C2—C1—C10	113.98 (14)	C11—C12—H12B	109.7
O1—C1—H1A	108.3	H12A—C12—H12B	108.2
C2—C1—H1A	108.3	C12—C13—C17	118.52 (14)
C10—C1—H1A	108.3	C12—C13—C14	106.96 (13)
C3—C2—C1	113.03 (15)	C17—C13—C14	101.66 (13)
C3—C2—H2A	109.0	C12—C13—C18	111.13 (13)
C1—C2—H2A	109.0	C17—C13—C18	105.30 (13)
C3—C2—H2B	109.0	C14—C13—C18	113.11 (13)
C1—C2—H2B	109.0	C15—C14—C8	120.04 (13)
H2A—C2—H2B	107.8	C15—C14—C13	105.81 (13)
O2—C3—C4	122.16 (16)	C8—C14—C13	112.52 (13)
O2—C3—C2	122.65 (16)	C15—C14—H14	105.8
C4—C3—C2	115.09 (15)	C8—C14—H14	105.8
C5—C4—C3	124.00 (16)	C13—C14—H14	105.8
C5—C4—H4	118.0	O3—C15—C16	112.84 (14)
C3—C4—H4	118.0	O3—C15—C14	109.39 (13)
C4—C5—C6	119.13 (16)	C16—C15—C14	102.90 (13)
C4—C5—C10	123.80 (16)	O3—C15—H15	110.5
C6—C5—C10	117.04 (14)	C16—C15—H15	110.5
C5—C6—C7	113.48 (13)	C14—C15—H15	110.5
C5—C6—H6A	108.9	O4—C16—C17	59.63 (10)
C7—C6—H6A	108.9	O4—C16—C15	113.00 (13)
C5—C6—H6B	108.9	C17—C16—C15	109.33 (14)
C7—C6—H6B	108.9	O4—C16—H16	120.1
H6A—C6—H6B	107.7	C17—C16—H16	120.1
C8—C7—C6	110.73 (14)	C15—C16—H16	120.1
C8—C7—H7A	109.5	O4—C17—C16	58.65 (10)
C6—C7—H7A	109.5	O4—C17—C20	111.69 (13)
C8—C7—H7B	109.5	C16—C17—C20	120.89 (14)
C6—C7—H7B	109.5	O4—C17—C13	115.34 (13)
H7A—C7—H7B	108.1	C16—C17—C13	107.21 (13)
C14—C8—C7	111.60 (13)	C20—C17—C13	125.04 (14)
C14—C8—C9	108.89 (13)	C13—C18—H18A	109.5
C7—C8—C9	110.14 (13)	C13—C18—H18B	109.5
C14—C8—H8	108.7	H18A—C18—H18B	109.5
C7—C8—H8	108.7	C13—C18—H18C	109.5
C9—C8—H8	108.7	H18A—C18—H18C	109.5
C11—C9—C8	113.17 (14)	H18B—C18—H18C	109.5
C11—C9—C10	113.43 (13)	C10—C19—H19A	109.5
C8—C9—C10	112.33 (12)	C10—C19—H19B	109.5
C11—C9—H9	105.7	H19A—C19—H19B	109.5
C8—C9—H9	105.7	C10—C19—H19C	109.5
C10—C9—H9	105.7	H19A—C19—H19C	109.5
C5—C10—C1	108.00 (13)	H19B—C19—H19C	109.5
C5—C10—C19	108.31 (13)	O5—C20—C21	121.64 (16)
C1—C10—C19	110.15 (13)	O5—C20—C17	120.23 (15)
C5—C10—C9	107.70 (13)	C21—C20—C17	118.12 (15)
C1—C10—C9	111.07 (13)	C20—C21—H21A	109.5
C19—C10—C9	111.47 (13)	C20—C21—H21B	109.5
C12—C11—C9	115.02 (13)	H21A—C21—H21B	109.5
C12—C11—H11A	108.5	C20—C21—H21C	109.5
C9—C11—H11A	108.5	H21A—C21—H21C	109.5
C12—C11—H11B	108.5	H21B—C21—H21C	109.5
C9—C11—H11B	108.5		
			
O1—C1—C2—C3	−177.45 (14)	C7—C8—C14—C15	−53.53 (19)
C10—C1—C2—C3	−55.2 (2)	C9—C8—C14—C15	−175.33 (13)
C1—C2—C3—O2	−153.53 (16)	C7—C8—C14—C13	−179.01 (13)
C1—C2—C3—C4	30.1 (2)	C9—C8—C14—C13	59.20 (17)
O2—C3—C4—C5	−174.79 (17)	C12—C13—C14—C15	160.68 (13)
C2—C3—C4—C5	1.6 (2)	C17—C13—C14—C15	35.75 (15)
C3—C4—C5—C6	168.03 (15)	C18—C13—C14—C15	−76.65 (17)
C3—C4—C5—C10	−9.8 (3)	C12—C13—C14—C8	−66.43 (16)
C4—C5—C6—C7	133.18 (16)	C17—C13—C14—C8	168.64 (12)
C10—C5—C6—C7	−48.82 (19)	C18—C13—C14—C8	56.24 (18)
C5—C6—C7—C8	51.31 (19)	C8—C14—C15—O3	77.47 (18)
C6—C7—C8—C14	−178.18 (13)	C13—C14—C15—O3	−153.96 (13)
C6—C7—C8—C9	−57.10 (17)	C8—C14—C15—C16	−162.36 (14)
C14—C8—C9—C11	−47.05 (17)	C13—C14—C15—C16	−33.78 (16)
C7—C8—C9—C11	−169.73 (13)	C17—O4—C16—C15	99.68 (15)
C14—C8—C9—C10	−177.10 (13)	O3—C15—C16—O4	71.90 (17)
C7—C8—C9—C10	60.23 (17)	C14—C15—C16—O4	−45.87 (16)
C4—C5—C10—C1	−13.9 (2)	O3—C15—C16—C17	136.24 (14)
C6—C5—C10—C1	168.22 (13)	C14—C15—C16—C17	18.47 (17)
C4—C5—C10—C19	105.41 (18)	C16—O4—C17—C20	113.86 (15)
C6—C5—C10—C19	−72.49 (17)	C16—O4—C17—C13	−95.44 (15)
C4—C5—C10—C9	−133.91 (16)	C15—C16—C17—O4	−105.92 (14)
C6—C5—C10—C9	48.19 (17)	O4—C16—C17—C20	−98.04 (15)
O1—C1—C10—C5	166.20 (12)	C15—C16—C17—C20	156.03 (14)
C2—C1—C10—C5	45.07 (19)	O4—C16—C17—C13	109.63 (13)
O1—C1—C10—C19	48.08 (17)	C15—C16—C17—C13	3.71 (17)
C2—C1—C10—C19	−73.04 (18)	C12—C13—C17—O4	−77.88 (18)
O1—C1—C10—C9	−75.92 (17)	C14—C13—C17—O4	38.94 (16)
C2—C1—C10—C9	162.96 (14)	C18—C13—C17—O4	157.10 (13)
C11—C9—C10—C5	176.74 (13)	C12—C13—C17—C16	−140.75 (14)
C8—C9—C10—C5	−53.34 (17)	C14—C13—C17—C16	−23.94 (16)
C11—C9—C10—C1	58.67 (18)	C18—C13—C17—C16	94.23 (14)
C8—C9—C10—C1	−171.41 (13)	C12—C13—C17—C20	68.4 (2)
C11—C9—C10—C19	−64.58 (17)	C14—C13—C17—C20	−174.80 (14)
C8—C9—C10—C19	65.34 (17)	C18—C13—C17—C20	−56.64 (19)
C8—C9—C11—C12	45.42 (18)	O4—C17—C20—O5	146.18 (15)
C10—C9—C11—C12	174.91 (13)	C16—C17—C20—O5	−148.31 (16)
C9—C11—C12—C13	−51.89 (18)	C13—C17—C20—O5	−1.1 (2)
C11—C12—C13—C17	173.60 (13)	O4—C17—C20—C21	−33.4 (2)
C11—C12—C13—C14	59.64 (17)	C16—C17—C20—C21	32.2 (2)
C11—C12—C13—C18	−64.26 (17)	C13—C17—C20—C21	179.35 (15)

### Hydrogen-bond geometry (Å, º)

**Table:**

*D*—H···*A*	*D*—H	H···*A*	*D*···*A*	*D*—H···*A*
O1—H1···O4^i^	0.88 (2)	2.06 (2)	2.929 (2)	168
O3—H3···O5^ii^	0.86 (2)	1.95 (2)	2.767 (2)	159
C1—H1*A*···O3^i^	1.00	2.59	3.527 (2)	157
C6—H6*A*···O1^ii^	0.99	2.59	3.527 (2)	158
C12—H12*B*···O3^iii^	0.99	2.52	3.468 (2)	161
C21—H21*B*···O2^iv^	0.98	2.53	3.501 (2)	170

Symmetry codes: (i) *x*+1/2, −*y*+3/2, −*z*; (ii) *x*−1, *y*, *z*; (iii) *x*+1, *y*, *z*; (iv) *x*, *y*−1, *z*.
